# Chronic Stress Decreases Cerebrovascular Responses During Rat Hindlimb Electrical Stimulation

**DOI:** 10.3389/fnins.2015.00462

**Published:** 2015-12-23

**Authors:** Sohee Lee, Bok-Man Kang, Min-Kyoo Shin, Jiwoong Min, Chaejeong Heo, Yubu Lee, Eunha Baeg, Minah Suh

**Affiliations:** ^1^Center for Neuroscience Imaging Research, Institute for Basic ScienceSuwon, South Korea; ^2^Department of Biological Science, Sungkyunkwan UniversitySuwon, South Korea; ^3^Department of Biomedical Engineering, Sungkyunkwan UniversitySuwon, South Korea; ^4^Department of Health Sciences and Technology, Samsung Advanced Institute for Health Sciences & Technology, Sungkyunkwan UniversitySeoul, South Korea

**Keywords:** chronic stress, sensory stimulation, neurovascular coupling, optical intrinsic signals, restraint stress, somatosensory cortex

## Abstract

Repeated stress is one of the major risk factors for cerebrovascular disease, including stroke, and vascular dementia. However, the functional alterations in the cerebral hemodynamic response induced by chronic stress have not been clarified. Here, we investigated the *in vivo* cerebral hemodynamic changes and accompanying cellular and molecular changes in chronically stressed rats. After 3 weeks of restraint stress, the elicitation of stress was verified by behavioral despair in the forced swimming test and by physical indicators of stress. The evoked changes in the cerebral blood volume and pial artery responses following hindpaw electrical stimulation were measured using optical intrinsic signal imaging. We observed that, compared to the control group, animals under chronic restraint stress exhibited a decreased hemodynamic response, with a smaller pial arterial dilation in the somatosensory cortex during hindpaw electrical stimulation. The effect of chronic restraint stress on vasomodulator enzymes, including neuronal nitric oxide synthase (nNOS) and heme oxygenase-2 (HO-2), was assessed in the somatosensory cortex. Chronic restraint stress downregulated nNOS and HO-2 compared to the control group. In addition, we examined the subtypes of cells that can explain the environmental changes due to the decreased vasomodulators. The expression of parvalbumin in GABAergic interneurons and glutamate receptor-1 in neurons were decreased, whereas the microglial activation was increased. Our results suggest that the chronic stress-induced alterations in cerebral vascular function and the modulations of the cellular expression in the neuro-vasomodulatory system may be crucial contributing factors in the development of various vascular-induced conditions in the brain.

## Introduction

Chronic stress, which is a common and unavoidable causal factor in many disease conditions, is believed to affect multiple body systems, including the cardiovascular system. Rapid delivery of nutrients and the removal of harmful chemicals through a local elevation of blood circulation play crucial roles in maintaining normal physiology. Malfunctions in the blood circulation are implicated in brain vascular diseases, including stroke, vascular dementia, and vascular parkinsonism (Diniz et al., 2013; Iadecola, 2013; Shih et al., 2013; Bauer et al., 2014; Korczyn, 2015). Harmonious interactions between neuronal and endothelial cells, enzymes and chemicals, must be maintained to ensure normal blood circulation, and it has been suggested that stress impairs this balance in various ways (Bryan, 1990; Black and Garbutt, 2002; Balkaya et al., 2011).

An *ex vivo* study showed that exposure to variable repeated stress and administration of corticosterone for 7 days lowered the responsiveness of the parenchymal arteriole in the amygdala to electrical stimulation (Longden et al., 2014). Stress also reduced the capillary diameters (Danielyan, 2008) and blood flow (Ohata et al., 1981; Lasbennes et al., 1986; Rahman et al., 2014) in several areas of the cortex in a rat acute or chronic immobilization stress model. In an electrophysiological study with brain slices, chronic stress decreased the function of inwardly rectifying K^+^ channels in the endothelial smooth muscle cells of the amygdala, followed by decreased responsiveness of the intracerebral arterioles upon electric field stimulation (Longden et al., 2014). In addition, stress impaired the modulation of vasomodulators. Molecular and histological studies showed that the activity of nitric oxide synthase (NOS), which catalyzes the production of nitric oxide (NO; a major gaseous vasodilator), was altered in a stress model (Duchemin et al., 2012; Tishkina et al., 2012; Gao et al., 2014).

A relatively small number of studies have examined the functional vascular changes induced by chronic stress in the brain *in vivo*. Human functional MRI studies showed the increment of peripheral arterial blood pressure and perfusion signal changes in several brain areas when the subjects perceived mild stress (Carter et al., 2005; Wang et al., 2005). Compared to non-stressed animals, the stressed animals showed lower cerebral blood flow changes in the somatosensory cortex elicited by inhalation of high concentrations of CO_2_ in a functional MRI study (Rahman et al., 2014). Because of the multi-faceted effects of chronic stress on hemodynamics, from modulator signaling to functional modulation, and the little direct evidence concerning the effect of chronic stress on the functional hemodynamic responses, the exact mechanisms responsible for the stress-evoked systematic changes remain elusive.

In this study, we, therefore, questioned the effect of chronic stress on behavior and cerebral blood perfusion *in vivo*, along with the accompanying vasomodulatory effects. We measured the real-time changes in the blood volume and vessel reactivity in the somatosensory cortex to the hindlimb sensory stimuli in chronically stressed rats using the *in vivo* optical imaging of intrinsic signals (OIS) technique. The OIS system can measure the blood perfusion changes with optical filters that have a specific wavelength to detect light reflected from oxy/deoxy hemoglobin (Suh et al., 2005; Vazquez et al., 2010). This allowed us to determine the real-time dynamic states of neurovascular coupling with high spatial and temporal resolution, but without any special dyes. To determine the factors that underlie the stress-induced decreased hemodynamic response, we also measured several representative vasomodulators and the proportion of cellular subtypes that are important to build the molecular environment. Our particular aim is to elucidate the systematic effect of chronic stress on cerebral perfusion and the vascular reactivity during sensory processing and to then correlate them to the underlying molecular changes in vasomodulatory signaling.

## Materials and methods

### Animal care

Eight-week old male Sprague-Dawley rats (OrientBio Inc., Seongnam, Korea) were used in this study. The animals were housed in pairs with *ad libitum* access to food and water. The environment was maintained with a 12-h light/dark cycle, 23–24°C temperature, and 50–60% humidity. Up on arrival, each animal underwent a 1-week adaptation period before the chronic stress modeling procedure. All experimental processes were approved by the Institutional Animal Care and the Use Committee (IACUC) of Sungkyunkwan University.

#### Chronic stress modeling

**Figure 1:**
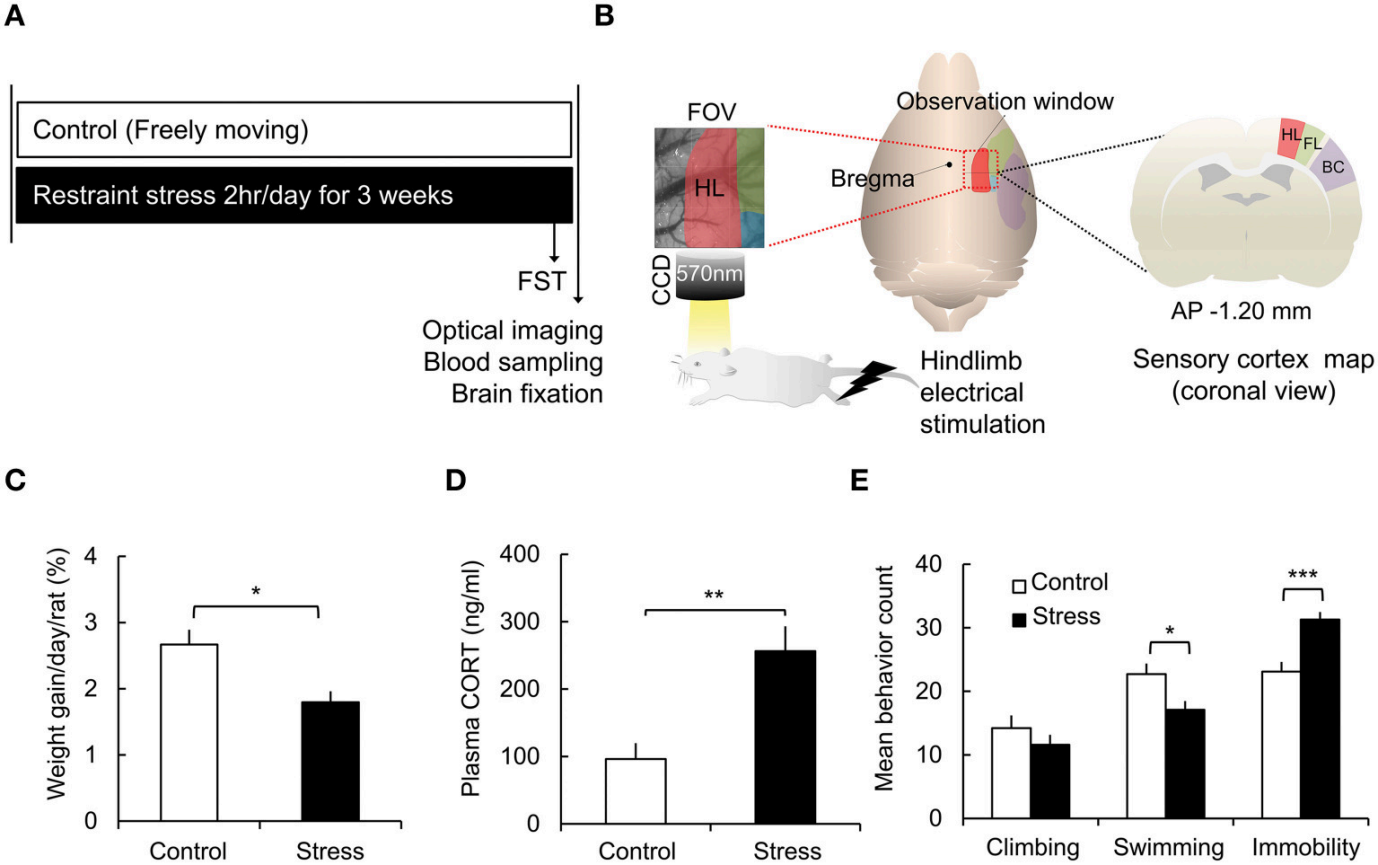
**Schematic of the experimental design and chronic stress model verification. (A)** Experimental protocol. **(B)** Acquisition of optical intrinsic signal imaging and field of view of the rat sensory cortex [red: hindlimb area (HL), green: forelimb area (FL), blue: shoulder area, purple: barrel cortex (BC)]. **(C)** Comparisons of the weight gain in the control group and the stress group [Control (*n*) = 10 and Stress (*n*) = 10]. **(D)** Analysis of corticosterone concentrations in the plasma [Control (*n*) = 10 and Stress (*n*) = 10]. **(E)** Behavioral analysis of the forced swimming test [FST, Control (*n*) = 10 and Stress (*n*) = 10]. The Mann–Whitney test and independent *t*-test were performed to analyze the significant differences (^*^p < 0.05; ^**^p < 0.01; ^***^p < 0.001).

The rats were immobilized with plastic bags (Decapicones, Braintree Scientific Inc., MA) in their home cages for 2 h each day. The stress procedure began at a set time (10:30 A.M.–12:30 P.M.) and was maintained for 3 weeks (Figure 1A). In additional experiments, the animals were exposed to acute stress (single 2-h stress) or 6-week stress (Supplementary Figure 1) to confirm the effects of single stress or more sustained stress respectively. During the restraint procedure, the animals were restricted from food and water intake. The rats in the control group were allowed to move freely in their home cages. The weight and food intake of the animals were monitored daily before stress exposure.

#### Behavioral test

To measure the behavioral despair resulting from chronic stress, the rats were subjected to a forced swimming test (FST) 1 day prior to the end of the chronic stress procedure (Control, *n* = 10; 3-week stress, *n* = 10). The FST was performed daytime (16:00–18:00 P.M.) several hours after completion of restraint stress. Because the swimming test itself could be an acute stressor even for the control group, we randomly chose one rat from each control cage for the FST. The control rat's behavior was considered to be representative of unstressed behavior, and the selected animal was excluded from the *in vivo* functional optical imaging study. The rats were placed in a cylindrical glass container (diameter 24.5 cm, height 36 cm) filled with 23 cm deep water (22–23°C) for 300 s. The water was freshly changed for each experiment. After testing, the rats were dried with paper towels and returned to their home cages. Each animal's behavior during the FST was recorded for 300 s with a camera, and the recording was divided into 60 epochs (5 s) for behavioral analysis. We classified the behaviors into three representative types of movements: climbing, swimming, and immobility. Climbing behavior was defined as perpendicular movement to the surface of water and was considered to be an active coping response, whereas the immobility behavior was defined as a non-voluntary and non-active movement in which the animal kept only its head above the surface of the water. Swimming behavior was defined as a horizontal movement across the surface. The predominant behavior was determined in each epoch.

### Functional assessment of chronic stress with real-time *in vivo* optical imaging and hindpaw electrical stimulation

The optical intrinsic signal imaging during direct hindpaw electrical stimulation were recorded 2 h after the last stress cessation (Control, *n* = 10; 3-week stress, *n* = 10; Figure 1A). Additional optical imaging data was collected in acute stress and 6-week stress group (Acute stress, *n* = 6; 6-week stress, *n* = 6, Supplementary Figure 1) with same imaging protocols of 3-week stress groups. For the *in vivo* experiment, the rat's head was fixed on a stereotaxic frame (David Kopf Instruments, Tujunga, CA, USA), and the animal was kept under general anesthesia with 1.5% isoflurane. We checked frequently the animal's arousal by tail pinch test and often increased the isoflurane concentration up to 1.8% for stabilizing anesthetized state. The rat's body temperature was maintained at 37–38°C using a homothermic heating pad controller (FHC Inc., ME, USA) with a rectal sensing probe, and its physiological status was monitored (PhysioSuite, Kent Scientific Corp, CT, USA). For real-time *in vivo* imaging, the animals underwent a full craniotomy, targeting the hindlimb area of somatosensory cortex. We removed a 5 × 7 mm^2^ piece of skull over the somatosensory cortex at area centered at −1.2 mm anterior-posterior (AP) and +3.5 mm medial-lateral (ML) from the bregma. After the craniotomy, the exposed cortex was illuminated with an LED lamp (CLS 150, Leica Microsystems CMS GmbH, Mannheim, Germany), and the functional cerebral blood volume changes were recorded with an optical imaging system (Imager 3001–Celox, Optical Imaging Ltd., Rehovot, Israel). The images were collected for 20 s using a fast acquisition camera (Photonfocus AG, Lachen, Switzerland) through 50 mm tandem lenses and a 570 ± 10 nm bandpass filter, as 570 nm is an isosbestic wavelength of oxyhemoglobin and deoxyhemoglobin (Figure 1B). A trial was composed of 200 frames acquired at a 10 Hz frequency.

To elicit the hemodynamic responses in the somatosensory cortex, a 1 mA, 5-s electrical stimulation was delivered to the hindpaw via 30-gauge needle electrodes (Grass Products, WI, USA). The electrical stimulation consisted of monophasic square pulses with a 5 ms pulse width at 3 Hz, which were generated by a nine-channel programmable stimulation generator (Master-9, A.M.P.I., Jerusalem, Israel) and were delivered 5 s after the onset of optical image acquisition. The inter-trial interval was 100 s, and the recordings were repeated 8 times.

### Blood sampling and enzyme-linked immunosorbent assay (ELISA)

The blood plasma corticosterone level was analyzed to verify the validity of our model. Both the control and stress groups (Control, *n* = 10; 3-week stress, *n* = 10) were briefly anesthetized with 3% isoflurane immediately after termination of the last stress, and the cardiac blood was collected in heparin-coated tubes (BD Vacutainer, Becton Dickinson, NJ, USA). The blood samples were centrifuged at 3500 rpm for 15 min. The segregated plasma was diluted 1:200 and analyzed using a corticosterone ELISA kit (Assaypro LLC, MO, USA). The fluorescence intensity was scanned at 450 nm by a microplate luminescence reader (Synergy HT, BioTek, VT, USA). A corticosterone standard curve was generated using standard solutions, and then, the sample concentration was determined from the standard curve.

#### Western blotting

Western blotting was performed (Control, *n* = 3; 3-week stress, *n* = 3) as previously described (Shin et al., 2014). Briefly, the proteins from the somatosensory cortex were extracted with a lysis buffer (50 mM Tris-Cl, 150 mM NaCl, 0.5% Triton X-100, 0.5% NP-40, and 1 mM EDTA) containing a complete protease inhibitor cocktail (Roche, Indianapolis, IN, USA) and phosphatase inhibitors (0.01 M Na_3_VO_4_ and 0.02 M NaF). The homogenates were centrifuged, and the protein concentrations of supernatants were determined by the Bradford assay (Bio-Rad, Hercules, CA, USA). Fifteen micrograms of the total proteins were boiled in Laemmli sample buffer. The proteins were separated by 8–15% sodium dodecyl sulfate/polyacrylamide gel electrophoresis and transferred to nitrocellulose membranes. The membranes were blocked in 5% non-fat dry milk/0.05% TBST for 1 h and incubated with the primary antibody overnight at 4°C. The primary antibodies included rabbit anti-neuronal NOS (nNOS; 4231S, Cell Signaling, MA, USA, 1:2000 dilution), rabbit anti-inflammatory NOS (iNOS; ab15323, Abcam, MA, USA, 1:2000 dilution), mouse anti-endothelial NOS (eNOS; 610297, Becton Dickinson, NJ, USA, 1:2000 dilution), mouse anti-HO-1 (ab12220, Abcam, MA, USA, 1:2000 dilution), rabbit anti-HO-2 (ab90515, Abcam, MA, USA, 1:2000 dilution), and rabbit anti-β-actin (A300-491A, Bethyl Laboratories, TX, USA, 1:2000 dilution). The membrane was subsequently incubated with horseradish peroxidase (HRP)-conjugated secondary antibodies. Finally, the blots were developed with a chemiluminescent HRP substrate kit (WBKLS0500, Millipore, MA, USA). The intensity of the bands was determined using ImageJ software (National Institutes of Health, MD, USA) and normalized to the β-actin values.

#### Immunohistochemistry

The rats (Control, *n* = 7; 3-week stress, *n* = 7) were anesthetized by inhalation of 3% isoflurane and were perfused with saline through a heart catheter, followed by 4% paraformaldehyde (PFA) infusion using a peristaltic pump (P-1500, Harvard apparatus, MA, USA). The brains were extracted and fixed in 4% PFA for 1 day and were then immersed in 30% sucrose in phosphate-buffered saline (PBS) for 3 days. The brains were frozen in tissue freezing medium (FSC22, Leica Biosystems, Wetzlar, Germany) and sectioned at 40 μm in the coronal direction using a cryostat (CM1950, Leica Biosystems, Wetzlar, Germany). The brain slices contained hindlimb areas at −1.2 to −1.5 mm AP from the bregma, which corresponded to the field of view (FOV) in the ORIS acquisition. The primary antibodies used in the present study were mouse anti-Parvalbumin (PV; P3088, Sigma-Aldrich, MO, USA, 1:2000 dilution), rabbit anti- Neuropeptide Y (NPY; 11976, Cell signaling, MA, USA, 1:1600 dilution), rabbit anti-glutamate receptor 1 (GluR-1, AMPA subtype; ab31232, Abcam, MA, USA, 1:1600 dilution), and rabbit anti-ionized calcium binding adaptor molecule-1 (Iba-1: 019-19741, Wako Chemicals, VA, USA, 1:400 dilution), followed by secondary antibody treatment. The secondary antibodies were anti-mouse Alexa-488 (A-21202, Molecular probes, OR, USA, 1:200 dilution) and anti-rabbit Alexa-568 (A-11042, Molecular probes, OR, USA, 1:200 dilution). After the antibody staining procedures, a mounting solution (H-1500, Vector Laboratory, CA, USA) containing 4′,6-diamidino-2-phenylindole (DAPI) was applied to the sections, and they were covered with a cover glass. The fluorescent images were acquired using confocal laser scanning microscopy (TCS SP8, Leica Microsystems CMS GmbH, Mannheim, Germany) with white light lasers and a macro confocal microscope (TCS LSI, Leica Microsystems CMS GmbH, Mannheim, Germany). The average number of the cells in 1 mm^2^ of bilateral hindlimb area was counted by ImageJ (National Institutes of Health, MD, USA) and custom-written Matlab (MathWorks, MA, USA) software with careful visual inspection.

### Analysis of cerebral blood volume change

To quantify the change in the cerebral blood volume (CBV), the acquired images were stored and analyzed with ImageJ and custom-written MATLAB software. To improve the signal-to-noise ratio, eight trials were averaged. And then, every pixels of an averaged image was filtered over frame with a low-pass filter for removing high-frequency components which are not evaluated as hemodynamic response, such as respiration and moving artifacts. A hemodynamic response map was obtained by dividing each image by a baseline (the averaged value of the initial 50 frames), and the images were cropped to 3 × 3 mm^2^, which contained the activation area responding to the hindpaw stimulation. We calculated the maximum CBV change in an automatically selected region of interest (ROI; 7 × 7 pixels) and calculated the time-to-peak activation by comparing every averaged value from consecutive three frames. The spatial extent of the activation area showing a more than 1.0% change of CBV from the baseline was calculated and was compared to the mean value of the baseline.

#### Vascular function analysis

To investigate the effect of stress on the vessels' response to sensory stimulation, we further studied the functional changes in the pial arteries and parenchymal tissues. First, we visualized the vascular pattern within the FOV using a trainable Waikato Environment for Knowledge Analysis (WEKA) segmentation plugin, which combines a collection of machine learning algorithms in Fiji (Hall et al., 2009; Schindelin et al., 2012). We classified the raw images into either vessel or tissue regions using the WEKA plugin, with a set of selected image features to produce pixel-based segmentation. This method employs the random forest classifier. Among all images, the images from four animals in the control group and five animals in the stress group were excluded in the following cases during the WEKA segmentation process: low contrast between the vessel and the tissue and micro hemorrhage on the cortical surface. Then, we set the threshold value at 60% of the probability for vessel classification after generating the vessel probability map for each pixel. This optimal threshold value was a tradeoff between the detection ratio and a false-positive ratio determined through an empirical analysis.

Next, we defined the “responsive area” in the FOV using a standard deviation (SD) map, which provides the degree of variation in a pixel's value over time. The SD map was computed from the pixel-by-pixel standard deviation of the total *N* frames within a data set as follows:

(1) $$\begin{array}{rcl} PA_{ij} & = & \frac{1}{N}\overset{N}{\underset{k=1}{\sum }}P_{ij}\left(k\right), \end{array}$$

(2) $$\begin{array}{rcl} SD_{map} & = & {\left\{\frac{1}{N}\overset{N}{\underset{k=1}{\sum }}{\left(P_{ij}\left(k\right)-PA_{ij}\right)}^{2}\right\}}^{1∕2}, \end{array}$$

where, *P*_*ij*_(*k*) denotes the value of a pixel located at the *i*th row and *j*th column in *k*th frame; *PA*_*ij*_ can be calculated by averaging value of *N* pixels obtained from the *i*th row and *j*th column in each frame. All maps were filtered by a 3 × 3 Gaussian kernel to prevent the signals from interfering with the outliers. The pixels in the SD map with a low level of variation were excluded from the responsive area because low variation suggests no response over time. The exclusion criterion was fixed by the threshold (mean + 2 SD) in the SD map. The pial artery was defined by multiplying the segmented vessel probability map with the masking image. We divided four characteristic regions through the process of image segmentation: the responsive area, the pial artery within the responsive area, the non-responsive vessels in the FOV, and the responsive tissue within the responsive area without the pial arteries. The intensity and dilation from the vessels, tissue and responsive area were assessed and averaged in every 3 frames. In the analysis of the hemodynamic response and vasodilation changes, the onset was defined as the moment where the value exceeded more than mean value plus two standard deviations of the baseline, and the rising slope was also calculated by measuring the incline ratio between the onset and the peak point.

### Statistics

Either a two-tailed Independent *t*-test or a Mann–Whitney test was performed for two independent group comparisons. To analyze more than two groups, One-way analysis of variance (ANOVA) was used with LSD *post-hoc* analysis. The data were expressed as the means ± standard error of the means (SEM). Statistical significances in the figures were indicated at one of three levels: ^*^p < 0.05, ^**^p < 0.01, ^***^p < 0.001.

## Results

### Effect of chronic stress on weight gain, corticosterone secretion, and behavior

To assess the effect of chronic stress on the animals' physical condition, weight gain, and corticosterone levels were measured. The weight gain of each animal was normalized to its initial weight. The rats in the stress group showed much less weight gain than the control group (Control, 2.67 ± 0.22%; Stress, 1.80 ± 0.16%, *p* = 0.0156 by the Mann–Whitney test; Figure 1C). The plasma corticosterone levels were significantly elevated in the stress group compared to the control group (Control, 96.19 ± 23.54 ng/ml; Stress, 256.27 ± 36.80 ng/ml, *p* = 0.002; Figure 1D).

The FST is a commonly used test to measure behavioral despair in a stress-induced depression model (Cryan et al., 2005; Voorhees et al., 2013). In the non-escapable water tank, the animals showed vigorous struggling, an active coping strategy, and then showed immobile floating behavior, reflecting a passive coping strategy, which was increased in enervated animals. In the present study, the chronic stress group showed decreased swimming behavior (Control, 22.7 ± 1.7; Stress, 17.1 ± 1.4, *p* = 0.019) and increased immobility behavior (Control, 23.1 ± 1.5; Stress, 31.3 ± 1.2, *p* = 0.001) compared to the control group (Figure 1E).

### Effect of hindpaw electrical stimulation on the hemodynamic responses

**Figure 2:**
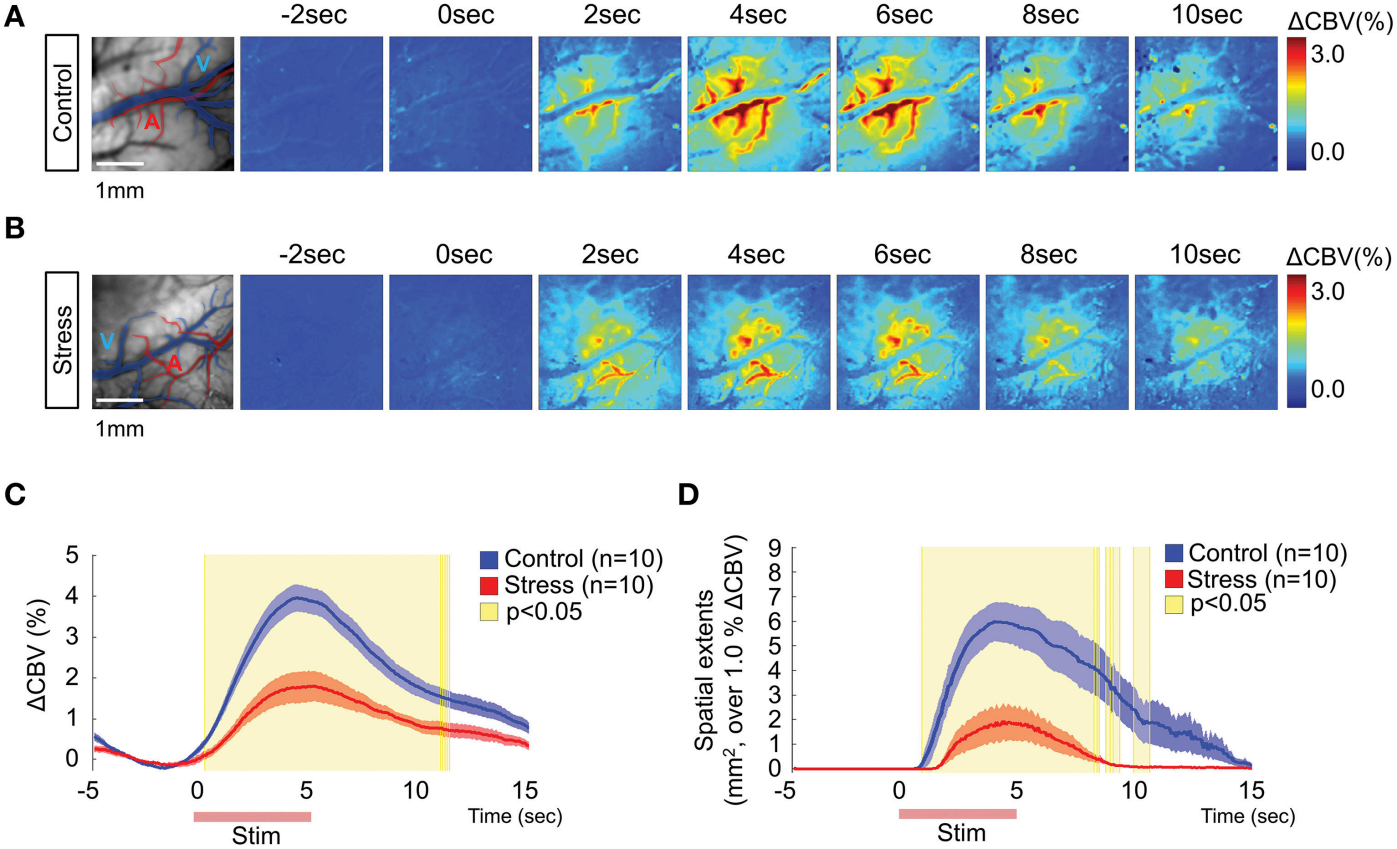
**Hemodynamic responses in the somatosensory cortex upon 1 mA hindpaw electrical stimulation**. Representative images of the hemodynamic responses from a non-stressed rat **(A)** and a 3-week chronically stressed rat **(B)**. The activation maps show the changes in the cerebral blood flow every other second following hindpaw electrical stimulation using color mapping. **(C)** The time series analysis of maximum ΔCBV in the control and stressed groups. **(D)** The time series analysis of the spatial extent of changes surpassing 1% ΔCBV. The red lines on the x-axis in **(C,D)** indicate the hindpaw stimulation period. The yellow-shaded areas on the plots of **(C,D)** represent the frames that showed significant changes between the control group and the stress group [Control (*n*) = 10 and Stress (*n*) = 10, Mann–Whitney test, *p* < 0.05].

Intrinsic optical signal imaging was used to assess the functional hemodynamic changes in the hindpaw area of the somatosensory cortex. Dividing all frames by the mean baseline frame (calculated over the 5 s prior to electrical stimulation) showed localized changes in the hindpaw area in the somatosensory cortex. The branches of superficial arteries were highly activated, while the pial veins did not show noticeable changes (Figures 2A,B). To better characterize how the hemodynamic signal changed over time, we selected a region of interest (ROI, 7 × 7 pixels) from which we could measure the maximum CBV change (Figure 2C). In the control group, the CBV started changing immediately after stimulation onset and peaked at 4.65 ± 0.25 s, with a maximum CBV change of 4.02 ± 0.34% compared to the 5 s baseline period. In contrast, the stress group showed a much reduced CBV response (1.82 ± 0.32%) that peaked at 5.43 ± 0.59 s. Significant differences in CBV between control and stress groups are indicated as yellow-shaded areas in Figure 2C. Additionally, we measured the spatial extents of the cortical area that was activated by the stimulation by setting a threshold to 1% of the CBV change compared to baseline. The stress group exhibited a much smaller CBV responsive area in the sensory cortex (Figure 2D). The acute stress group exhibited higher hemodynamic responses (2.99 ± 0.18%, *n* = 6) than 3-week stress group (*p* = 0.026), but tended to decreased hemodynamic response in comparison to control group (*p* = 0.053; Supplementary Figure 1). We also confirmed the reduced hemodynamic response in a smaller responsive area from the prolonged chronic restraint stress group (6-week stress, *n* = 6), which occurred even with stronger electrical stimulation (Supplementary Figure 1). Taken together, these observations indicate that the hemodynamic function is reduced in the stressed animals.

### The influence of chronic stress on the vascular dynamics

**Figure 3:**
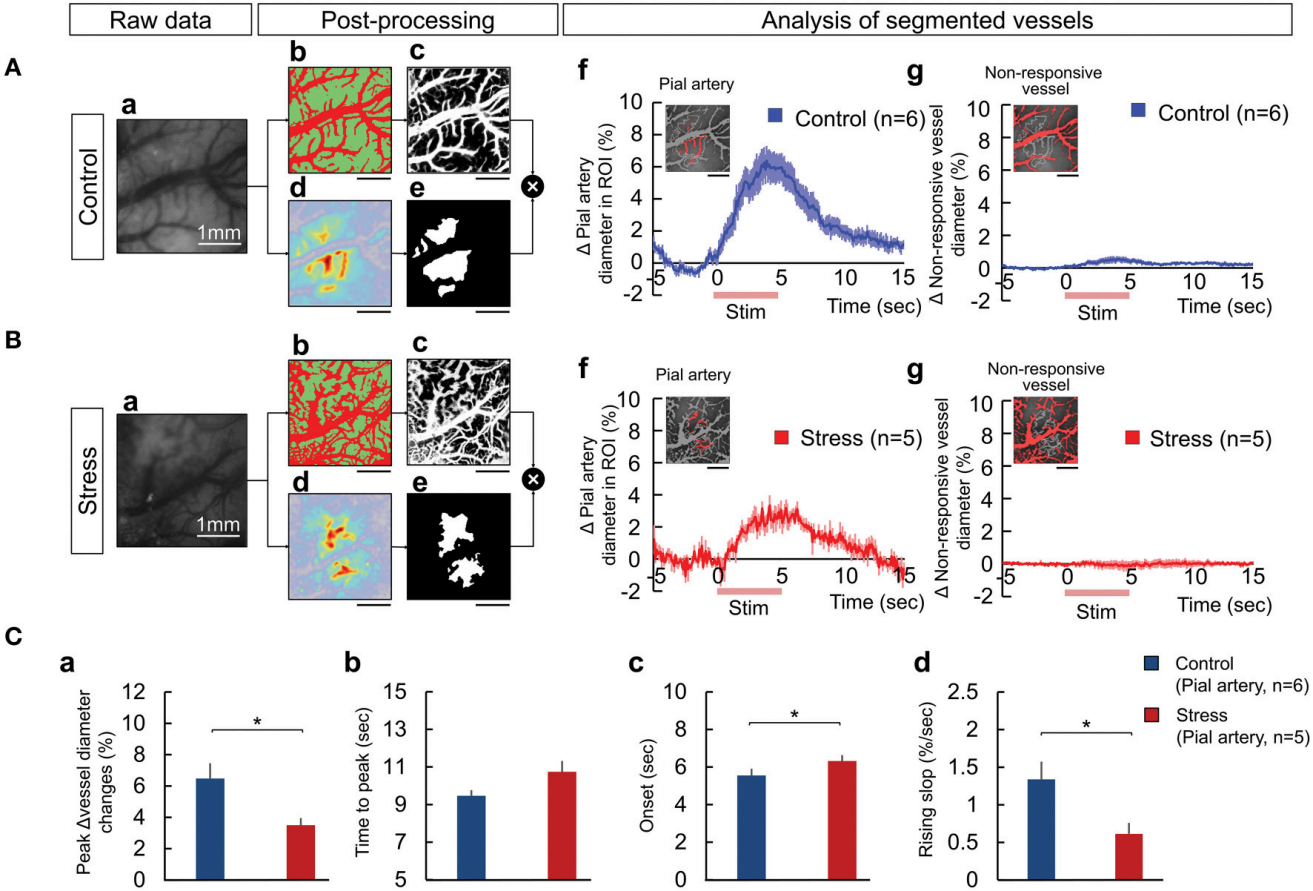
**The process of vessel segmentation used to extract the responsive pial artery to measure the hemodynamic response upon peripheral somatosensory stimulation in the control (A) and 3-week stress (B) groups**. The responses of the vessels were displayed as the changes in the number of pixels over the threshold, a standard for distinguishing the vessels from the tissues. The vessel segmentation was performed by combining a Weka segmentation-training method (image J) and standard deviation mapping: **(Aa,Ba)** raw image, **(Ab,Bb)** classified images of the vessel and tissue using Weka segmentation method in the raw image (the red region represents the vessel and the green region represents the tissue), **(Ac,Bc)** vessel probability map, **(Ad,Bd)** a color-coded standard deviation map (SD map), **(Ae,Be)** the mask image used to define the “responsive area” based on the SD map, **(Af,Bf)** the responsive pial artery defined by the mask image overlapping the vessel probability map and the dynamics of the responsive pial arterial diameter, and **(Ag,Bg)** the non-responding vessels upon electrical stimulation and dynamic changes of the vessel diameters. The red lines on the x-axis in **(Af,g,Bf,g)** indicate the hindpaw stimulation period. All scale bars under the representative segmentation images indicate 1mm. **(C)** Comparisons of the dynamics between the pial artery of the control and stress groups in the maximum vessel dilation change **(Ca)**, the time to maximum peak **(Cb)**, the onset time **(Cc)**, and the rising slope **(Cd)** [Control (*n*) = 6 and Stress (*n*) = 5, Independent *t*-test, ^*^p < 0.05].

The temporal hemodynamic responses to hindpaw stimulation between the vessels and tissue were explored to understand the source of the decreased responses observed in the stress group. We segmented the vessels from the responsive area of OIS analysis, using vessel segmentation and SD mapping (see Materials and Methods for detail). Figures 3Ab,Bb and Ac,Bc illustrate the classified vessel (red) and tissue (green) images and the computed vessel probability map respectively from raw images (Figures 3Aa,Ba). The responsive area was automatically calculated by SD mapping (Figures 3Ad,Bd), which provides the threshold for defining the responsive and non-responsive areas in the FOV (Figures 3Ae,Be). Then, we quantified the changes in the vascular diameter by counting the pixel changes of the selected vessels during the stimulation trial. The animals with chronic stress exhibited significantly lower diameter changes in the pial artery within the responsive area compared to control group (Figures 3Af,Bf,Ca; Control, 6.48 ± 2.38%; Stress, 3.49 ± 1.02%, *p* = 0.029). In contrast, the vessels in the non-responsive area (Figures 3Ag,Bg) rarely showed any pixel change in any of the animals (Control, 0.65 ± 0.41%; Stress, 0.30 ± 0.37%, non-significant differences). Figures 3Ca–d described the decreased pial arterial dynamic changes in chronic stress group compared to control group.

The average intensities of the entire responsive area, pial artery, and tissue were reduced in the stress group than the control group (Supplementary Figure 2A). We normalized each individual maximum value of the intensity changes to one hundred to observe the dynamic properties. For the inter-region discrimination in the control group, there was different time-to-peak response between the tissue and pial artery, as shown in Supplementary Figure 2Ca (Control, *p* = 0.041, by paired *t*-test). However, in the stress group, we could observe a diminished difference in the dynamic properties between the tissue and pial artery. When we evaluated the inter-group discrimination, the rising slope in the pial artery was lower (pial artery, *p* = 0.029) in the chronic stress group (Supplementary Figure 2Cb). Moreover, the 3-week stress group showed more micro hemorrhages on the dura mater and bled frequently than control group.

### Cellular and molecular effects of chronic stress

**Figure 4:**
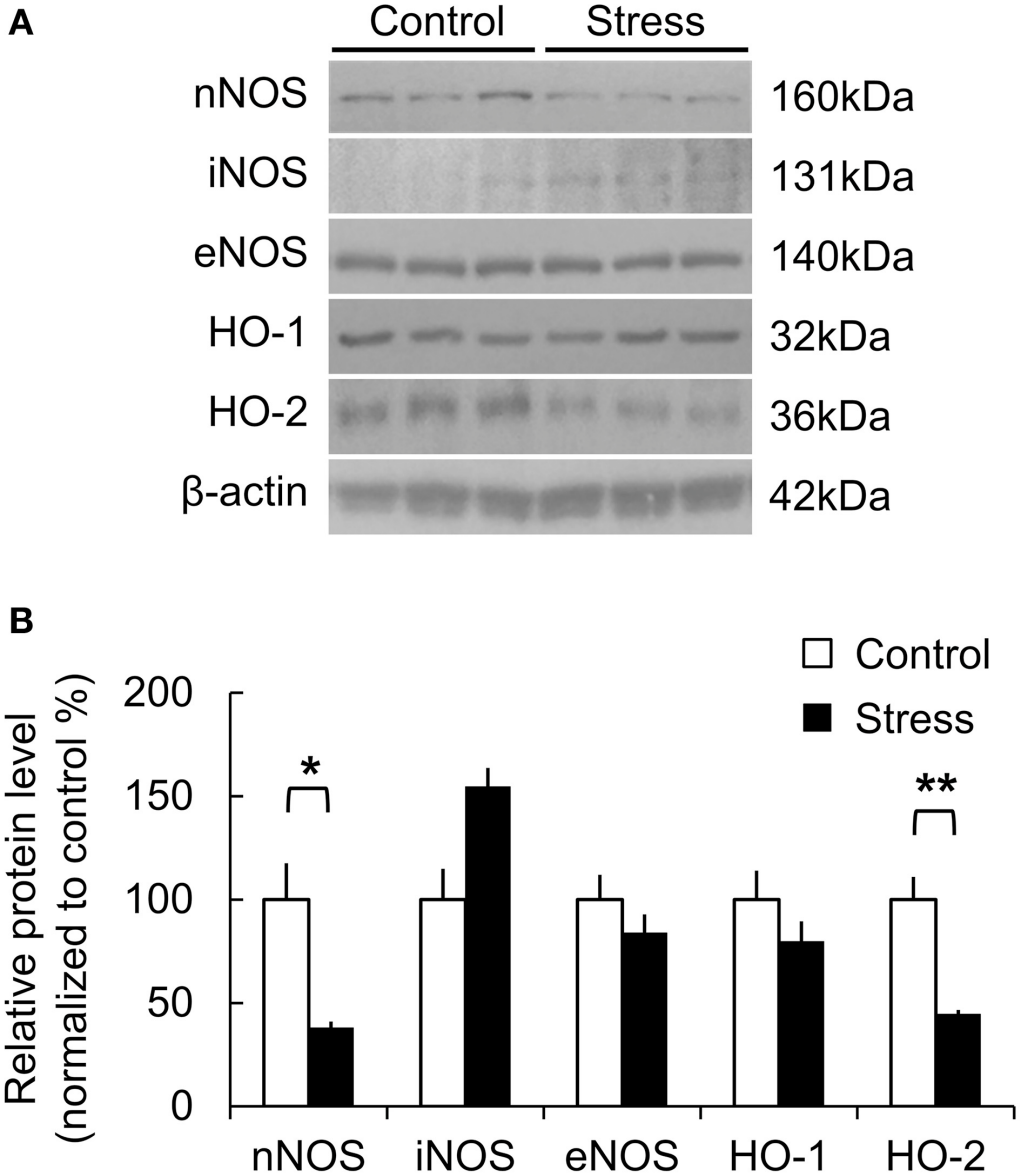
**Protein expression related to vasomodulation in the somatosensory cortex of control and 3-week stress groups**. **(A,B)** Changes in expression of NO- and CO-producing enzymes. The western blot band intensity was normalized to the control by densitometry [nNOS, neuronal nitric oxide synthase; iNOS, inflammatory nitric oxide synthase; eNOS, endothelial nitric oxide synthase; HO-1, Heme oxygenase-1; HO-2, Heme oxygenase-2, Control (*n*) = 3 and Stress (*n*) = 3, Independent *t*-test, ^*^p < 0.05; ^**^p < 0.01].

The molecular changes due to chronic stress were studied by analyzing the protein expression of the major vasomodulatory molecules in the somatosensory cortex, including the NO- and CO-producing enzymes that mediate vasodilation in the brain. NO is derived from neuronal NO synthase (nNOS), endothelial NO synthase (eNOS), or inflammatory NO synthase (iNOS). In the stress group, nNOS expression was significantly lower (Control, 100 ± 17.61%; Stress, 38.04 ± 3.00%, *p* = 0.047), while iNOS expression was higher than the control group (Control, 100 ± 14.95%; Stress, 154.65 ± 9.08%, *p* = 0.063). Inducible HO-1 and constitutive HO-2 generate the gaseous vasomodulator CO. The expression of HO-2 was significantly reduced in the stress group (Control, 100 ± 10.99%; Stress, 44.65 ± 2.03%, *p* = 0.008). HO-1 and eNOS expression did not show significant differences between the control and stressed brain cortices (Figures 4A,B).

**Figure 5:**
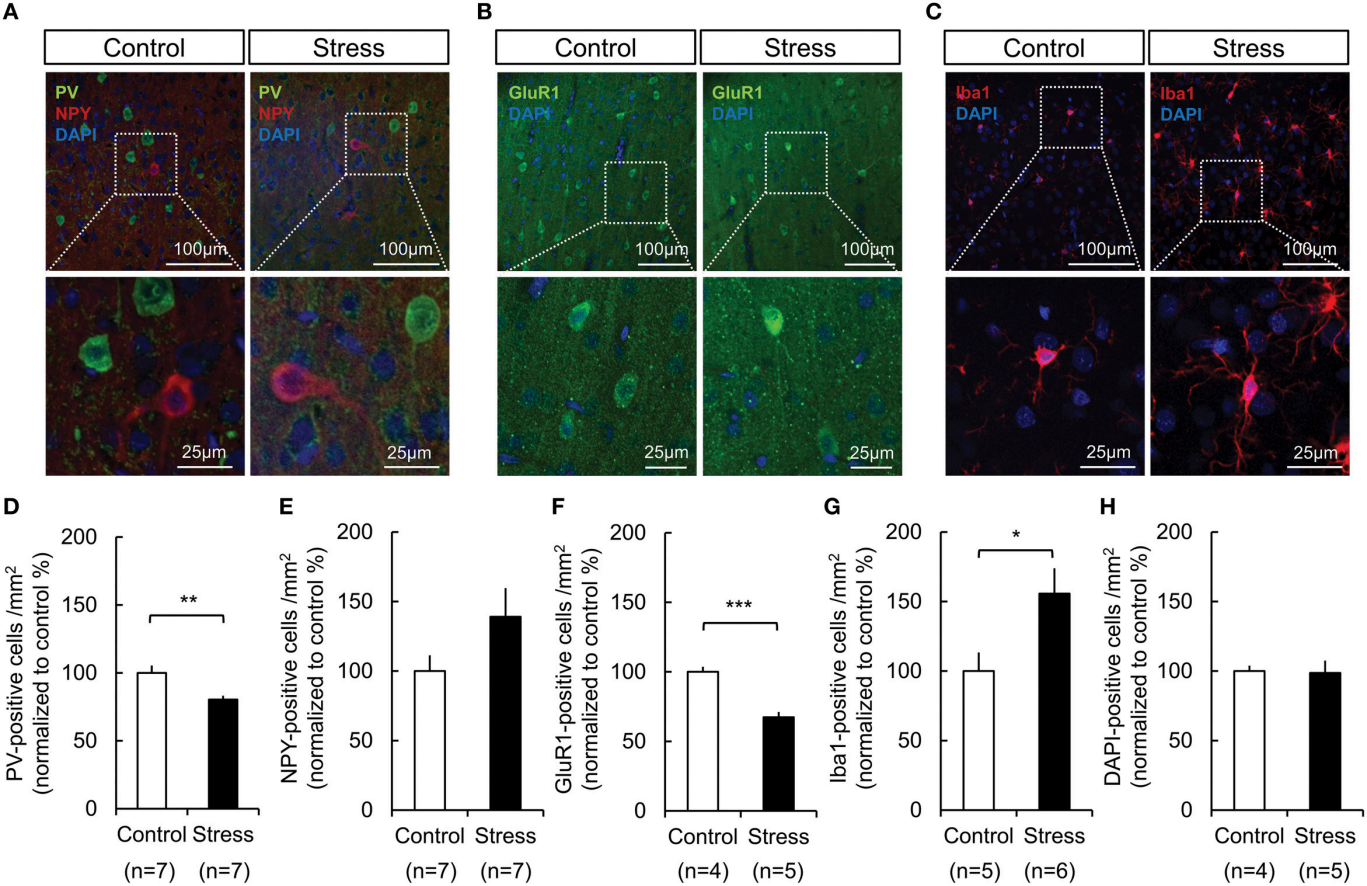
**Changes in the expression of PV in GABAergic interneurons, GluR-1 in neurons, and microglial activation in the somatosensory cortex of control and 3-week restraint-stressed rats**. **(A)** Representative images of PV-positive cells (green), NPY-positive cells (red), **(B)** GluR-1-positive cells (green), and **(C)** Iba1-positive microglial cells (red). All nuclei are stained using DAPI. The images in the white dashed box are magnified in the lower image. The number of PV- labeled cells **(D)**, NPY- labeled cells **(E)**, GluR1- labeled cells **(F)**, Iba1- labeled cells **(G)**, and DAPI- labeled nuclei **(H)** in both animal groups were counted (PV, parvalbumin; NPY, neuropeptide Y; DAPI, 4′,6-diamidino-2-phenylindole; GluR1, glutamate receptor 1; Iba1, ionized calcium binding adaptor molecule 1, Independent *t*-test, ^*^p < 0.05; ^**^p < 0.01; ^***^p < 0.001).

Immunohistochemical analysis was performed to study the cellular subtypes involved in the response, which included GABAergic interneurons, ionotropic GluR-1-immunoreactive cells, and microglial activation. The analysis was restricted to the hindlimb areas of the somatosensory cortex. As shown in Figure 5 the PV expression in GABAergic interneurons (Control, 100 ± 5.45%; Stress, 80.40 ± 2.76%, *p* = 0.008) and glutamate receptor-1 expression in neurons (Control, 100 ± 3.67%; Stress, 67.39 ± 3.79%, *p* = 0.001) were significantly decreased, whereas the NPY expression in GABAergic interneurons tended to be increased in the chronically stressed rat cortex. Additionally, microglial activation was increased (Control, 100 ± 13.33%; Stress, 155.67 ± 18.26%, *p* = 0.042) under chronic stress conditions. The total cell number, which was determined by counting of DAPI-stained nuclei, did not show significant changes between unstressed and stressed animals.

## Discussion

The present study characterizes the functional hemodynamic responses in the stressed brain *in vivo*. Chronic stress reduced normal functional perfusion to an external stimulus, as shown by the diminished cerebral blood volumes and pial arterial dilation in the somatosensory cortex following hindlimb stimulation. In addition, we examined the cellular and molecular changes underlying the reduced hemodynamics in the stress group. Stress reduced the vasodilator producing enzymes, including nNOS and HO-2, and changed the cellular subpopulations that are believed to be part of the neurovascular unit. This study suggests an extensive range of stress-induced effects are produced in the cerebrovascular system.

### Reduced hemodynamic responses during sensory processing in chronic stressed animals

There are few studies that have attempted to understand the effects of stress on the vascular system in the brain. This is partly because most stress research has focused on the cellular and molecular effects of glucocorticoids, due to activation of the HPA axis (Vyas et al., 2002; Herman et al., 2003; Roozendaal et al., 2009; Ulrich-Lai and Herman, 2009). Moreover, previous studies have investigated the stress-induced changes in several brain regions, such as the hippocampus, amygdala, hypothalamus, and prefrontal cortex (Herman et al., 2003; Hill et al., 2012; Mcklveen et al., 2013). Prolonged stress, however, can produce systematic effects on the brain as a whole through the cerebrovascular system. Using the widely used chronic stress paradigm for 3 weeks (Cook and Wellman, 2004; Schnell et al., 2012; Musazzi et al., 2015), we found that the hemodynamic responses to hindpaw electrical stimulation were significantly reduced in stressed animals. The observed stress-induced reductions in the hemodynamic responses in the somatosensory cortex naturally imply an extensive effect of chronic stress on the brain, although different areas in the brain can be affected differently. In addition, the results from the stress condition may indicate reduced neuronal responses to external stimuli in the somatosensory cortex, when we accept the general notion of neurovascular coupling, which is a correlation between neural activity and cerebral blood flow recruitment. A combination of electrophysiology and functional neuroimaging may elucidate the effect of stress on neurovascular coupling in future studies.

Our results indicate that the source of the decreased functional hemodynamic responses to the hindpaw electrical stimulation may be related to the decreased reactivity of the pial arteries compared to the parenchymal tissue, as shown by the reduced dilation of the pial arteries during hindpaw stimulation. In addition, the stress group showed that blood volume recruitment via the pial artery was reduced and delayed compared to the control group, implying overall alterations to the cerebrovascular system, including arteries and capillaries. This effect may be related to the functional decline to the pial artery that was induced by reduced responsibilities of the cells in the artery (Bernatova and Csizmadiova, 2006; Custodis et al., 2011; Chen et al., 2014), a dysfunctional blood brain barrier (Esposito et al., 2001; Najjar et al., 2013), and other causes. Recent *ex vivo* studies showed that dysfunctional inwardly rectifying K^+^ channels in the endothelial smooth muscle cells of the stressed amygdala can be evidence of the decreased arterial function from our study (Longden et al., 2014). In any case, the stress-induced adverse effects to the pial arteries may be a crucial factor because the pial arteries are a key site that controls the flow to the intra-parenchymal arterioles that supply the activated area.

The acute stress effects may not be excluded in the present study because the imaging session was conducted several hours after last stress exposure. However, 3-week stress animals showed that more reduced hemodynamic responses in comparison to acute stress animals and less reduced hemodynamic responses than 6-week stress animals. This suggests there are cumulative effects of stress on vascular responsiveness. Further studies may be necessary to confirm the non-linearity of hemodynamic responses induced by the amount and degree of exposure to stress.

### Alterations in vasomodulatory system and cellular atmosphere by exposures to chronic stress

We sought to further understand the vasomodulator changes that link chronic stress and the systematic effects on cerebrovascular system. Our stress model induced enhanced iNOS expression and decreased nNOS and HO-2 expression, which could contribute to the stress-induced hemodynamic and vascular functional impairments. The increased iNOS expression may indicate changes in inflammation-mediated signaling pathways and increased neurotoxicity. The extensive and prolonged release of NO, which is induced by the increased iNOS expression after chronic immobilization stress (Olivenza et al., 2000), is, at least partially, responsible for the stress-induced pathological condition (Brown, 2007). As we will discuss later, the decreased number of PV-positive neurons and GluR1-positive neurons from our immunohistochemical approach would be the consequence of the iNOS-induced changes to neural cells.

The increased iNOS expression may also suggest that stress activates inflammatory mechanisms. We found an increased microglial activation, which exert cytotoxic effects by releasing inflammatory mediators, including iNOS (Boje and Arora, 1992; Possel et al., 2000). These results are consistent with studies showing that increases in microglial proliferation and ramification occur in the hippocampus and amygdala in response to chronic stress (Nair and Bonneau, 2006; Tynan et al., 2010). It has been known that repeated stress can elevate pro-inflammatory cytokines, such as IL-1β, IL-6, and TNF-α (You et al., 2011). These pro-inflammatory cytokines can affect microglial activation. Concurrently, microglial cells can release pro-inflammatory cytokines. The increased levels of pro-inflammatory cytokines in the brain exacerbate stress-induced pathological conditions, including the downregulated hemodynamics and vascular reactivity observed this study.

In addition to the iNOS increase, our observation that nNOS expression is decreased in stress group may also stem from the pathological process that is initiated by stress-induced inflammation. However, other studies have reported up-regulation of nNOS in the hypothalamic-pituitary-adrenal axis (Kishimoto et al., 1996), and in the amygdala and hippocampus (Echeverry et al., 2004) by relatively acute stress. In contrast, the same study showed that nNOS expression was not changed in the secondary somatosensory cortex (Echeverry et al., 2004). The resulting reduction of nNOS, which is aberrant in normal conditions, compared to iNOS in cortical pyramidal cells may be the main factor underlying the decrease in the functional hemodynamic and vascular responses in the stress group. In addition, the decrease in HO-2 expression, which produces CO, may contribute to the reduced functional hemodynamic responses in the stress group, although the role of CO in stress has not been reported. Recent evidence from an *in vivo* microsensor study demonstrates that CO has a tight relationship with increased metabolic demands and cerebral blood flow (Park et al., 2015). In this study, the endogenous CO release nicely reflects the robust hemodynamic response, which is induced by epidural electrical stimulation in the rat somatosensory cortex. Therefore, in our study, decreased HO-2 expression may give us a plausible explanation for the decreased hemodynamics.

**Figure 6:**
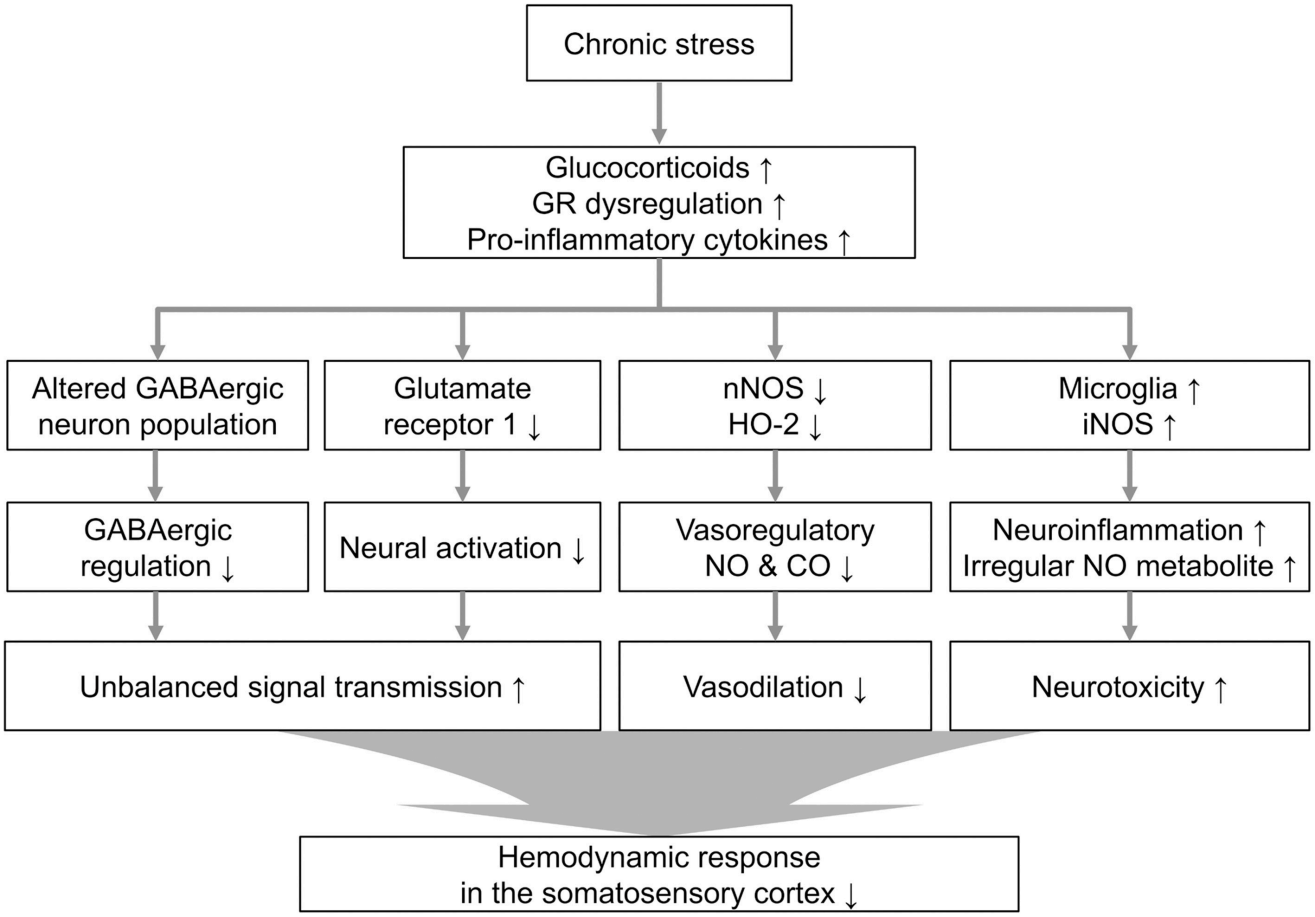
**Proposed cellular and molecular signaling pathway alterations associated with chronic stress**. Theoretical cascade of the effects of chronic stress, including the neurovasomodulatory alterations suggested by these experimental results from the restraint-stressed rats (GR, glucocorticoid receptor).

Our observation of downregulated vasomodulator expression in the stress group prompted us to investigate the cellular atmosphere that can reflect the stress-induced molecular changes. Sustained activation of the glucocorticoid receptor during chronic stress affected the network of PV-expressing GABAergic interneurons in the hippocampus (Heine et al., 2005). It was recently shown that maternal stress could decrease development of fetal GABAergic interneurons in the medial frontal cortex (mPFC) and hippocampus, and the effects of the stress spanned the neonatal PV-expressing interneuron population (Uchida et al., 2014). In addition, PV-positive interneurons are reportedly related to the modulation of vessel dilation. Although, the PV-positive interneurons are not included in the major vasomodulator-releasing neuronal subtypes (Perrenoud et al., 2012), NOS-releasing neurons do receive synaptic input from PV neurons (Morello et al., 1997), and there is a study reporting that a subtype of PV-expressing interneurons co-localizes with nNOS-immunoreactive cells (Liang et al., 2013). The decreased vessel dilation and the reduction of nNOS could be partly explained by the reduction of PV-expressing neurons in our immunohistochemical results, although the exact dynamics require further examination. Based on our observations, we modeled a process of neurovascular dysfunction in the chronically stressed brain in Figure 6. Chronic stress may induce changes in signal transduction through glucocorticoid and glutamate release. It has been intensively studied that stress-induced failure to down-regulate excitotoxic glutamate and pro-inflammatory cytokines is conducive to a neurotoxic environment (de Kloet et al., 2005; Cohen et al., 2012; Wood et al., 2015). Sustained stress can also affect the negative feedback of the stress-related endocrine system (Charmandari et al., 2005). In the present study, we also observed that chronic stress can lead changes in constitutive vasomodulators which play important role in regulation of vascular function during sensory signal transduction. Therefore, the decreased hemodynamics are compounded by the diverse consequences of the molecular and cellular reactions to chronic stress.

In summary, we show that chronic stress may affect the production of vasomodulators, such as NO and CO. These altered states of the vasomodulatory system in the stressed brain could lead to the decreased hemodynamic and vascular responses during hindpaw sensory processing. We also found altered cellular responses in the stressed brain, including down-regulation of expression of PV in GABAergic interneurons and GluR-1 in neurons, and an up-regulation of microglia activation. However, additional studies are needed to determine how excitatory neurons and specific subtypes of GABAergic interneuron may interact with the neurovascular coupling units under stressed conditions. It is also necessary to study the role of microglia in regulating cerebrovascular system during stress to understand the systematic effect of stress in the brain. Moreover, our results suggest that chronic stress may cause systematic changes in cerebrovascular function from the cellular to the functional level. Measurements for the reduced hemodynamic and vascular responses to sensory processing can be a good index to assess the effects of chronic stress at the molecular, cellular and system levels.

## Author contributions

MS and SL designed the study. SL, MS, JM, and CH performed research. SL, BK, and YL analyzed data. EB helped with manuscript writing. MS and SL wrote the paper.

### Conflict of interest statement

The authors declare that the research was conducted in the absence of any commercial or financial relationships that could be construed as a potential conflict of interest.
