# Supplementary material for: Chronic Stress Decreases Cerebrovascular Responses During Rat Hindlimb Electrical Stimulation
Source: Front Neurosci. 2015 Dec 23;9:462. doi: 10.3389/fnins.2015.00462 (PMC4688360; doi:10.3389/fnins.2015.00462)
Supplement: Supplementary file 1 [file Image1.pdf]

Supplementary Material

Chronic stress induces cerebrovascular dysfunction during rat hindlimb electrical stimulation

Sohee Lee[1], Bok-Man Kang[1], Min-Kyoo Shin[2], Jiwoong Min[3], Chaejeong Heo[1], Yubu Lee[1], Eunha Baeg[1], and Minah Suh[1,2,3,4]*

[1]Center for Neuroscience Imaging Research (CNIR), Institute for Basic Science (IBS), Suwon, Republic of Korea
[2]Department of Biological Science, Sungkyunkwan University, Suwon, Republic of Korea
[3]Department of Biomedical Engineering, Sungkyunkwan University, Suwon, Republic of Korea
[4]Department of Health Sciences and Technology, SAIHST, Sungkyunkwan University, Suwon, Republic of Korea
*** Correspondence:** Minah Suh: minahsuh@skku.edu

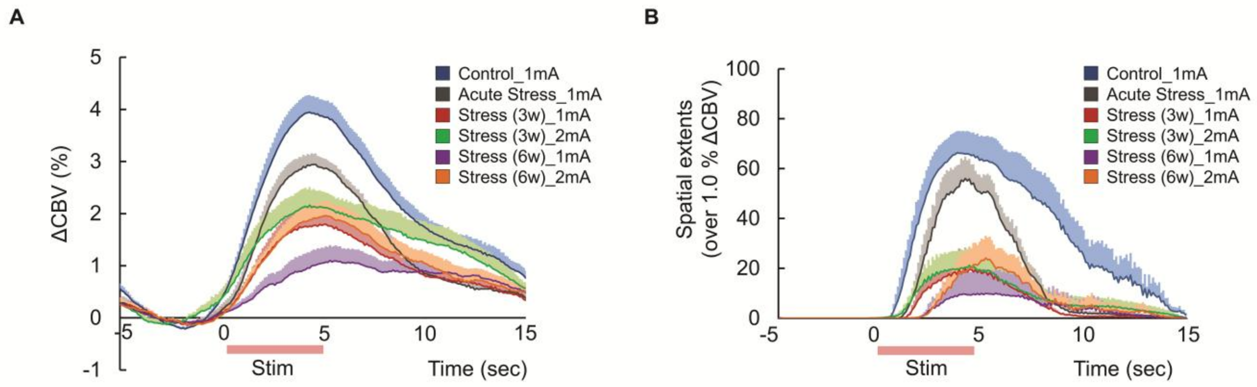

Supplementary Figure 1. Hemodynamic responses in the somatosensory cortex upon hindpaw electrical stimulation in the control, acute stress, 3-week stress, and 6-week stress groups. **(A)** Time series analysis of ΔCBV in selected ROI (7 × 7 pixels) showing the maximum intensity changes. The data from the control and 3-week stressed groups upon 1 mA stimulation were described in Fig. 2 in the main manuscript. The 6-week stressed group showed reduced cerebral blood flow recruitment compared to the control group, which was same as the 3-week stressed group in both the 1 mA and 2 mA stimulations. **(B)** Time series analysis of the spatial extent of the changes that were beyond 1 % ΔCBV. The red lines on the x-axis in **(A, B)** indicated the 5 sec hindlimb stimulation period (Control, n=10; Acute Stress, n=6; 3-week Stress, n=10; 6-week Stress, n=6).

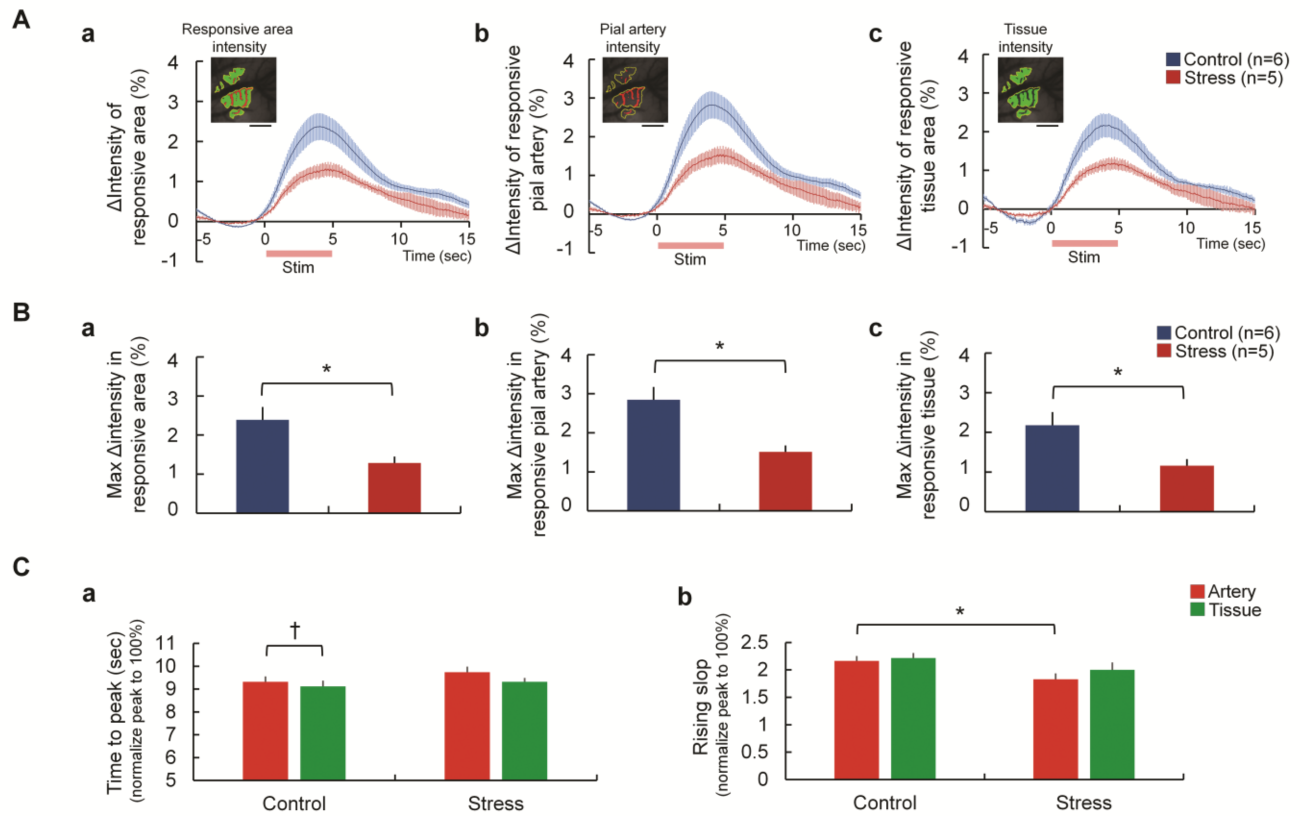

Supplementary Figure 2. The changes in the intensity **(A)** and hemodynamic properties **(B)** in the responsive area, pial artery, and tissue upon 1mA hindlimb stimulation in the control and 3-week stress groups. The regions were defined by the SD map and the vessel probability map (see additional details in the Materials and Methods). All scale bars under the representative segmentation images indicate 1 mm. Time series analysis of the averaged intensity and maximum Δ intensity in the responsive area **(Aa, Ba)**, pial artery **(Ab, Bb)**, and tissue **(Ac, Bc)**. **(C)** The hemodynamic properties in the tissue and pial artery of each group. The analyses were performed on the averaged values of the time to maximum peak **(Ca)** and the rising slope **(Cb)** (Control, n=6; 3-week Stress, n=5; *:$p<0.05$, independent *t*-test in inter-group comparisons; †:$p<0.05$ paired *t*-test in intra-individual comparisons).
